# cExternal beam radiation results in minimal changes in post void residual urine volumes during the treatment of clinically localized prostate cancer

**DOI:** 10.1186/1748-717X-4-26

**Published:** 2009-07-22

**Authors:** Peter F Orio, Gregory S Merrick, Zachariah A Allen, Wayne M Butler, Kent E Wallner, Brian S Kurko, Robert W Galbreath

**Affiliations:** 1 Brooke Army Medical Center Department of Radiation Oncology, Ft. Sam, Houston, TX 78234, USA; 2 Schiffler Cancer Center and Wheeling Jesuit University 1 Medical Park Wheeling, WV 26003, USA; 3 Puget Sound Healthcare Corporation Group Health Cooperative University of Washington Seattle, WA 98108, USA

## Abstract

**Background:**

To evaluate the impact of external beam radiation therapy (XRT) on weekly ultrasound determined post-void residual (PVR) urine volumes in patients with prostate cancer.

**Methods:**

125 patients received XRT for clinically localized prostate cancer. XRT was delivered to the prostate only (n = 66) or if the risk of lymph node involvement was greater than 10% to the whole pelvis followed by a prostate boost (n = 59). All patients were irradiated in the prone position in a custom hip-fix mobilization device with an empty bladder and rectum. PVR was obtained at baseline and weekly. Multiple clinical and treatment parameters were evaluated as predictors for weekly PVR changes.

**Results:**

The mean patient age was 73.9 years with a mean pre-treatment prostate volume of 53.3 cc, a mean IPSS of 11.3 and a mean baseline PVR of 57.6 cc. During treatment, PVR decreased from baseline in both cohorts with the absolute difference within the limits of accuracy of the bladder scanner. Alpha-blockers did not predict for a lower PVR during treatment. There was no significant difference in mean PVR urine volumes or differences from baseline in either the prostate only or pelvic radiation groups (p = 0.664 and p = 0.458, respectively). Patients with a larger baseline PVR (>40 cc) had a greater reduction in PVR, although the greatest reduction was seen between weeks one and three. Patients with a small PVR (<40 cc) had no demonstrable change throughout treatment.

**Conclusion:**

Prostate XRT results in clinically insignificant changes in weekly PVR volumes, suggesting that radiation induced bladder irritation does not substantially influence bladder residual urine volumes.

## Introduction

Increasingly sophisticated conformal radiotherapy delivery technologies and organ localization protocols have resulted in significant changes in treatment paradigms offered to patients with clinically localized prostate cancer. These technologies allow physicians to offer dose escalations to the targets while respecting normal tissue tolerances of surrounding organs [1-4]. Simultaneously, smaller treatment margins are employed to minimize side effects and potential complications. As a result, the precise evaluation of internal organ movement has become extremely important to ensure optimal dose to the target area. Three-dimensional conformal radiotherapy (3D-CRT) led to significant sparing of normal tissue by conforming the dose to the prostate gland. As a result, 3D-CRT was the first modality to generate widespread concern about prostate gland motion during treatment. Intensity modulated radiation therapy (IMRT) produces much steeper dose gradients than 3D-CRT and may result in tighter margins between the clinical target volume (CTV) and the planning target volume (PTV). Internal organ displacement of even a few millimeters may result in geographic miss of the target volume. Methods to monitor prostate motion have become increasingly important in the era of dose escalation. The use of computerized tomography (CT) has been the gold standard for in vivo imaging as well as structure identification, and has been emphasized in numerous internal organ motion studies [5-9]. Although cone beam CT has been integrated into linear accelerator systems, most CT studies are performed in a manner simulating treatment. For this reason, many institutions implant intraprostatic gold fiducial markers for identification on electronic portal imaging. This provides three dimensional information regarding prostate position in relation to the treatment isocenter [10,11]. Other technologies, such as the BAT ultrasound system and intraprostatic electromagnetic transponders are also solutions to account for daily variations in prostate positioning [12,13].

Variables with the potential to influence prostate motion are an important aspect of clinical research in the delivery and treatment of prostate cancer. The two organs receiving the greatest scrutiny are the bladder and rectum secondary to the close proximity to the prostate gland. The literature demonstrates a robust relationship between the influence of rectal filling on prostate displacement, where as the influence of the bladder is a little more controversial [6,7,9,14,15]. Researchers who have reported displacement of the prostate by the bladder have typically demonstrated movement to be in the posterior and inferior direction [6-8,10,16]. Conversely other researchers have reported no or a minimal influence of bladder filling on prostate motion [5,9,15,17,18]. Techniques in patient immobilization, treatment position and instructions to maintain a full or empty bladder during treatment may influence the bladder and prostate interaction [9,11,18].

This body of research specifically addresses the influence of daily whole pelvic or prostate only daily radiation treatments on weekly ultrasound determined post-void residual (PVR) urine volumes in patients with clinically localized prostate cancer treated prone with an empty bladder. This analysis helps to provide insight into PVR urine volume variations as patient's progress through radiation treatments to determine if such changes are clinically significant.

## Methods

One hundred and twenty five patients were treated for clinical stage T1b-T3a (2002 AJCC) prostate cancer with either definitive external beam radiation therapy to the prostate only (n = 68) or to the whole pelvis followed by a prostate boost (n = 59) [19]. For patients with < 10% risk of pelvic lymph node involvement, the target volume consisted of the prostate gland and seminal vesicles with margin [20]. For patients with > 10% risk of pelvic lymph node involvement, the pelvic lymph nodes were included in the initial target volume. Intensity Modulated Radiation Therapy (IMRT) was utilized in all treatments. Patient treated with prostate only radiation received 81 Gy. Patients who were treated with pelvic radiation received 45 Gy to the prostate and regional nodes followed by a 36 Gy boost to the prostate.

All patients were irradiated in the prone position and immobilized in a custom aquaplast hip-fix immobilization device with an empty bladder and rectum at the time of simulation and treatment. Patients were instructed to urinate immediately prior to initial CT simulation and daily during external beam radiation therapy. Patients were instructed to defecate prior to simulation and daily radiation if the urge was felt.

At the time of CT simulation, PVR volumes were measured within 10 minutes of voiding by transabdominal ultrasonography (Bladder Scan BVI 3000, Diagnostic Ultrasound, Brothel, Washington). PVR determinations were obtained weekly throughout treatment. These values were compared to the baseline PVR volume from the time of simulation. PVR urine volumes determined by ultrasound were not compared to CT scan as previous investigators have determined there is a high degree of correlation between bladder scanner volumes and Computed Tomography volumes and more importantly weekly changes from baseline were measured by ultrasound and not computed tomography [18,21-23]. Previously published correlations for the BVI model 3000 range from 0.86–0.95 [21,23]. The bladder scanner is reported to operate within a margin of accuracy of ± 20 cc in the range of 0 to 699 ml of urine volume. Accuracy of the bladder scanner is reported by the manufacturer within the operator's manual and as based on scanning diagnostic ultrasound tissue equivalent phantoms [24].

The BVI 3000 bladder scanner is a portable Ultrasound originally developed to measure residual urine volumes after micturition. The scanning head is positioned on the patient's body 2 cm above the pubic symphysis in a midline position. The bladder volume is calculated from a 2 MHz transducer which automatically rotates in 15 degree increments to provide a 3-dimensional model of the bladder to estimate the urine volume. Two highly experienced nurses, specifically trained and competent in the use of the BVI 3000, performed all scans analyzed in this study.

An alpha-blocker was initiated in 56 patients at a mean of 4.7 weeks ± 2.2 weeks into treatment. Alpha blockers were initiated for urinary irritative or obstructive symptoms. Alpha-blockers consisted of either tamsulosin hydrochloride (0.4 – 0.8 mg daily) or terazosin hydrochloride (5–10 mg daily).

One-way ANOVA, t-tests, and Fisher's exact chi-squared were applied to the clinical and treatment parameters of the two treatment cohorts (prostate only and pelvic radiation patients). All data was analyzed using SPSS version 14.0 software (SPSS, Inc., Chicago, IL). Statistical significance was set at a p < 0.05 for all analyses. In scatter plots of PVR over time, various empirical regression functions were tested for an optimum fit to the data, and either a quadratic function, y = a + b * time + c * time^2 ^or a logistic regression function, y = a/1+ b * exp ^-c * Time ^where y is either the PVR urine volume or the difference between the PVR urine volume and the baseline PVR urine volume, consistently outperformed linear regression by resulting in a larger correlation coefficient and therefore were used uniformly throughout.

## Results

Table 1 summarizes the clinical and treatment parameters of the study population, stratified by treatment cohort. The mean patient age was 73.9 ± 8.0 years with a mean pre-treatment prostate volume of 53.3 ± 33.5 cubic centimeters, a mean PVR urine volume of 57.6 ± 77.3 cubic centimeters. Patients treated with prostate only external beam radiation therapy compared with patients treated with whole pelvic radiation therapy had statistically lower pre-treatment PSA (p = 0.011); lower Gleason Score (p < 0.001); lower percent positive biopsies (p < 0.001; earlier staged disease (p < 0.001); a lower incidence of perineural invasion (p = 0.001) and were less likely to have received androgen deprivation therapy (ADT) (p < 0.001). No statistical differences were demonstrated between the groups concerning patient age at treatment, prostate volume, post-void residual urine (PVR) volumes and the use of alpha-blockers during treatment.

**Table 1:** Clinical and treatment parameters of the study population stratified by treatment cohort.

Continuous Variables		Prostate only (n = 66)		Pelvis (n = 59)			All Patients (n = 125)	
					
		Mean ± SD	Median	Mean ± SD	Median	p*	Mean ± SD	Median
Age at treatment (years)		73.9 ± 7.8	75.5	73.9 ± 8.3	76.3	0.971	73.9 ± 8.0	75.8
Pre-treatment IPSS		10.9 ± 7.0	10.0	11.8 ± 8.3	11.5	0.526	11.3 ± 7.6	10.0
Pre-treatment PSA (ng/mL)		7.7 ± 5.3	6.5	29.6 ± 68.4	10.3	0.011	18.0 ± 48.0	7.3
Gleason Score		6.5 ± 0.7	6.	7.8 ± 1.2	8.0	< 0.001	7.1 ± 1.2	7.0
% positive biopsies		29.0 ± 23.7	18.5	62.3 ± 32.8	62.5	< 0.001	44.2 ± 32.6	33.3
Prostate volume (cm^3^)		54.8 ± 33.8	47.3	51.6 ± 33.2	42.0	0.597	53.3 ± 33.5	46.5
Post void residual (cc)		57.3 ± 67.0	30.0	58.0 ± 88.0	27.0	0.961	57.6 ± 77.3	28.0
BMI		27.8 ± 3.9	27.4	29.2 ± 5.0	28.5	0.950	28.4 ± 4.4	28.2

Categorical Variables		Count (%)		Count (%)		*p*^∀^	Count (%)	

Stage (median)	T1b-T2b	65 (98.5)		46 (78.0)		**< 0.001**	111 (88.8)	
	≥ T2c	1 (1.5)		13 (22.0)			14 (11.2)	
ADT	none	55 (83.3)		19 (32.2)		**< 0.001**	74 (59.2)	
	≤ 6 months	7 (10.6)		2 (3.4)			9 (7.2)	
	> 6 months	4 (6.1)		38 (64.4)			42 (33.6)	
Diabetes		11 (16.9)		14 (23.7)		0.236	25 (20.0)	
Hypertension		43 (65.2)		39 (66.1)		0.531	82 (65.6)	
Alpha blocker		53 (80.3)		43 (72.9)		0.221	96 (76.8)	
Perineural invasion		13 (19.7)		28 (47.5)		**0.001**	41 (32.8)	

* *p *values calculated by one-way ANOVA

∀ *p *values determined by 2-sided Fisher's Exact Test

Of the 125 patients, 96 were exposed to alpha blockers during their treatment [Table 2, 3]. A total of 56 patients were placed on alpha blockers during treatment. Forty patients were actively treated with alpha-blockers prior to radiation. Thirty patients were started on alpha blockers at a mean of 4.70 ± 2.2 weeks in the prostate only group and twenty-six patients initiated alpha-blockers at a mean of 4.7 ± 2.4 weeks in the pelvic radiation group (p = 0.941) [Table 2].

**Table 2:** Week of alpha-blocker initiation relative to start of external beam therapy, stratified by radiation cohort.

XRT Therapy Cohort	Number. of patients*	Alpha-blocker Initiated (weeks)		*p*^†^
		
		Mean ± SD	Median	
Prostate Only	30	4.7 ± 2.2	4.5	0.941
Pelvis	26	4.7 ± 2.4	4.5	
Overall	56	4.7 ± 2.3	4.5	

* – Does not include patients who started alpha-blockers prior to treatment.

^† ^*p*-value determined by one-way ANOVA

**Table 3:** Variation in individual post-void residual (PVR) volume readings over the course of the study, stratified by therapy cohort, alpha-blocker use, baseline PVR volume group, and radiation cohort.

Parameter	Group	Number of patients	Mean of N weeks of PVR readings	*p*-value	Mean Std. Dev. of N PVR readings	*p*-value
					
			Mean ± SD		Mean ± SD	
Alpha-blocker use	No	29	26.5 ± 29.3	0.027	22.3 ± 18.8	0.011
	Yes	96	54.7 ± 65.6		35.7 ± 36.9	
Baseline PVR volume	≤ 40 cc	76	23.2 ± 31.7	**< 0.001**	23.4 ± 32.7	**<0.001**
	> 40 cc	49	86.9 ± 72.7		46.7 ± 31.4	
Radiation cohort	Prostate only	66	46.3 ± 55.4	0.725	29.7 ± 21.6	0.317
	Pelvis	59	50.2 ± 65.6		35.8 ± 43.9	
Overall population		125	48.2 ± 60.2		32.5 ± 34.0	

* The median number N of PVR readings was 10.

*p*-values were calculated by independent samples t-test.

Table 3 summarizes the variation in PVR urine volume readings over the course of the study, stratified by therapy cohort, alpha-blocker use, baseline PVR volume group and radiation cohort. Of the 125 patients included for analysis, 66 patients were treated with prostate only radiation and 59 patients were treated with whole pelvic radiation therapy followed by a prostate boost. Seventy-six patients had a PVR urine volume at the time of simulation measured to be less than or equal to 40 cc. Forty-nine patients had PVR urine volumes greater than 40 cc. For the overall population, the mean PVR urine volume over the entire course of radiation treatment was 48.2 cc. The mean individual PVR urine volume over all weeks of the study for patients with a baseline PVR > 40 cc was 86.9 cc verses in comparison to 23.2 cc in the patient group with baseline PVR ≤ 40 cc (p < 0.001). No significant difference was found between the mean individual PVR urine volume over all weeks of treatment in patients treated with prostate only versus pelvic radiation with values of 46.3 cc and 50.2 cc, respectively (p = 0.725).

**Figure 1:**
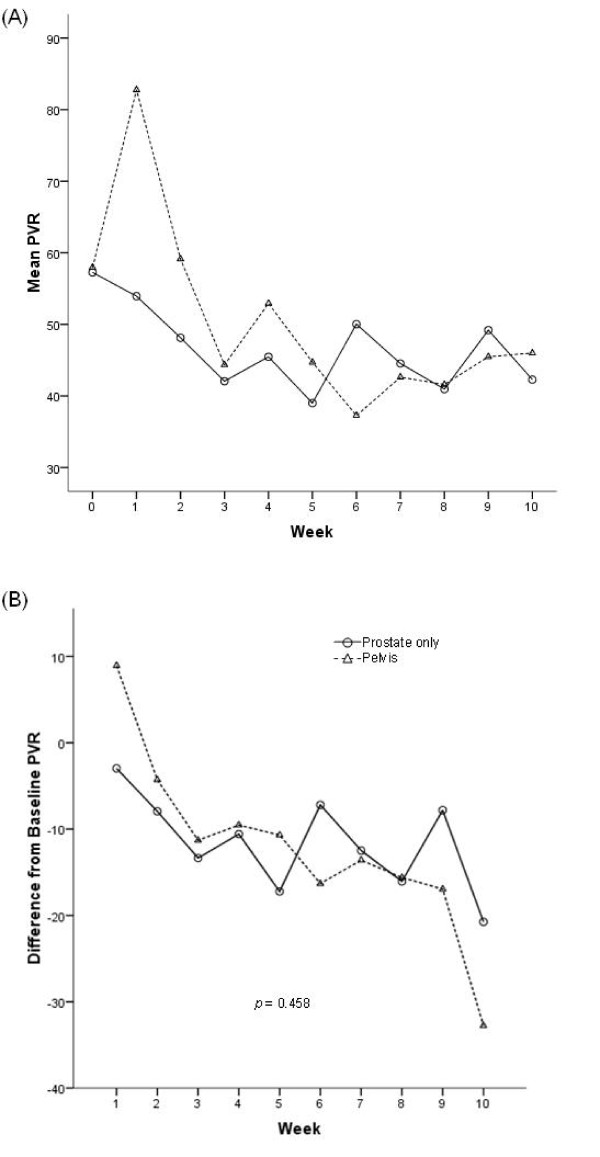
**(A). Mean post-void residual volume as a function of week of external beam radiation therapy (XRT) treatment, stratified by radiation group**. The bladder scanner operates within a margin of accuracy of ± 20 cc. **(B) **Mean difference from baseline in post-void residual (PVR) volume as a function of week of external beam radiation therapy (XRT) treatment, stratified by radiation group. The best-fit lines were determined by quadratic regression analysis. The bladder scanner operates within a margin of accuracy of ± 20 cc.

Figure 1a demonstrates that the mean PVR urine volume between the two treatment cohorts were not significantly different from each other (p = 0.664) over the duration of therapy. The mean PVR urine volumes demonstrated the greatest decreased over the first three weeks in both prostate only and pelvic radiation groups, although became variable with time and demonstrated an increase towards the end of therapy back to baseline measurements. The magnitude of difference is less than 20 cc in both cohorts, which are at the limit of accuracy of the bladder scanner. Figure 1b graphs the mean difference in baseline PVR urine volumes as a function of weeks of external beam radiation therapy. Both cohorts of patients demonstrated a decrease from baseline measurements with the greatest trend seen over the first three weeks of treatment. No significant differences were demonstrated concerning the magnitude of change from baseline PVR urine volumes when comparing pelvic radiation to prostate only radiation.

**Figure 2:**
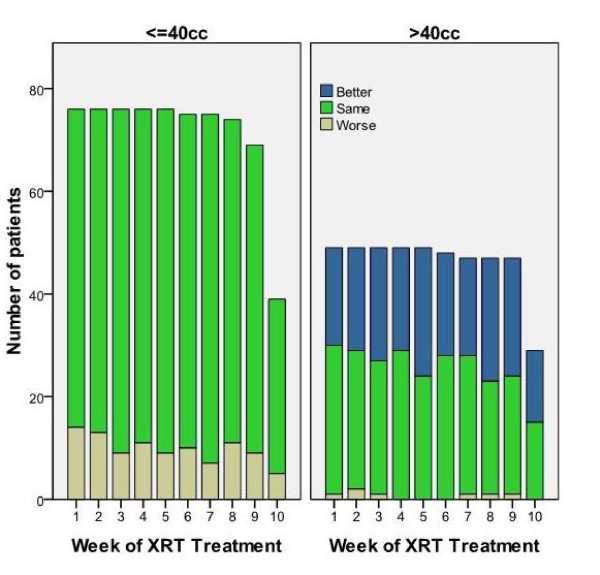
**Distribution of PVR cohort by week and stratified by pre-treatment post-void residual volume**. Patients moving from the lower PVR category (≤ 50 cc) to the higher category (> 50 cc) were labeled as worse, while patients in the higher category who moved to the lower were labeled as better. The number of patients in each baseline category varies over time based upon treatment length.

Larger baseline PVR allows for a greater absolute volume changes as radiation induced bladder irritability increases, therefore patients were stratified into two groups based on initial PVR. A cut-off of 40 cc was chosen as previous studies have demonstrated that bladder volumes greater than 40 cc in addition to rectal filling had the potential to influence daily prostate position while treated in the prone position [9]. Figure 2 shows the distribution of PVR cohort by week and is stratified by pre-treatment PVR ≤ 40 or > 40 cc. Patients were defined as having a worse PVR if they moved from a lower PVR category (≤ 40 cc) to the higher category (> 40 cc), while patients in the higher category who moved to a lower category were defined as better. During subsequent points in time, only a small fraction of patients with an initial PVR ≤ 40 cc exceeded 40 cc, while patients with an initial PVR > 40 cc had a high probability of a subsequent PVR < 40 cc.

**Figure 3:**
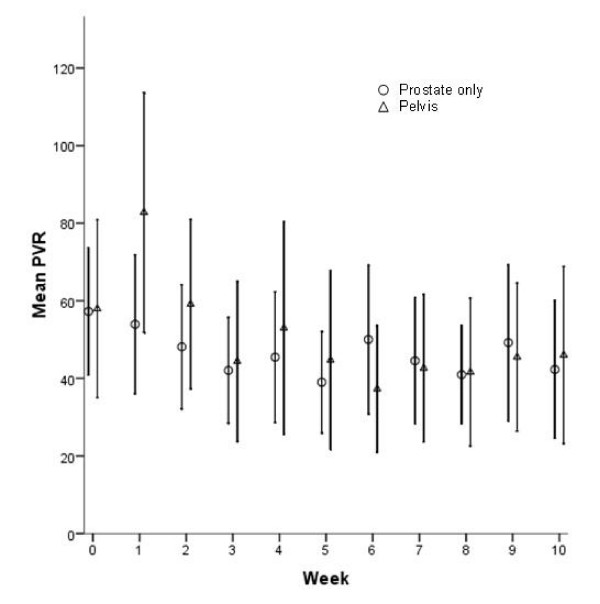
**Plot of mean post-void residual volume ± 2 standard error versus week of XRT treatment, stratified by pre-treatment (baseline) post-void residual volume**. The number of patients in each baseline category varies over time based upon treatment length.

Figure 3 graphically demonstrates the mean PVR urine volumes by week of radiation treatment for both prostate only and pelvic radiation patients to two standard deviations. Over two standard deviations the mean PVR reported are similar between the two groups albeit variable due to the intrinsic accuracy of ± 20 cc of the bladder scanner. The radiation field utilized did not appear to greatly influence the mean PVR compared to one another.

**Figure 4:**
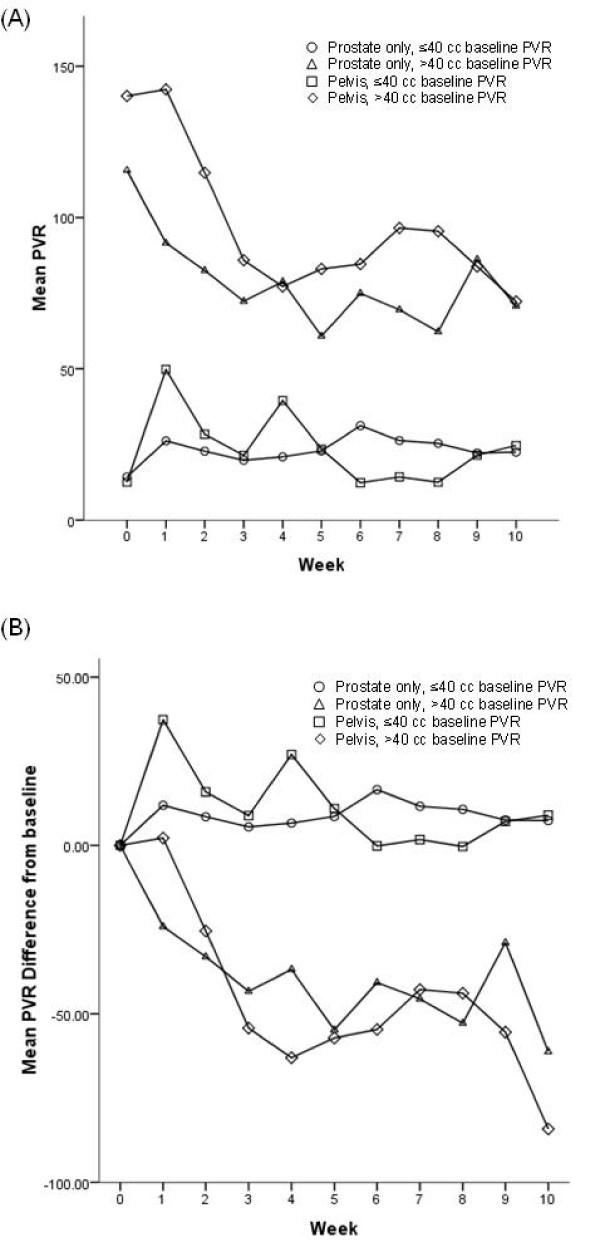
**(A) Plot of mean post-void residual volume versus week of XRT treatment, stratified by treatment group and pre-treatment (baseline) post-void residual volume**. The number of patients in each baseline category varies over time based upon treatment length. **(B) **Plot of mean difference from baseline in post-void residual volume versus week of XRT treatment, stratified by treatment group and pre-treatment (baseline) post-void residual volume. The number of patients in each baseline category varies over time based upon treatment length.

Figure 4a graphically represents the mean PVR urine volumes versus weeks of radiation treatment, stratified by treatment group and baseline PVR urine volumes ≤40 or >40 cc. The greatest changes over time in mean PVR were demonstrated in both treatment cohorts with base line volumes greater than 40 cc. As demonstrated in previous graphs the greatest and most consistent change is over the first three weeks of treatment. Very little change in the mean PVR is demonstrated in the group of patients treated with prostate only radiation with baseline PVR urine volumes less than or equal to 40 cc. The same is demonstrated in the pelvic radiation group with slightly greater variability, although within the limits of the bladder scanner. Figure 4b graphically represents the mean difference from baseline PVR urine volumes versus weeks of radiation treatment, stratified by treatment group and baseline PVR urine volumes <40 or >40 cc. The data continues to demonstrate very little change in PVR volumes over time for both treatment cohorts when baseline PVR urine volumes are less than or equal to 40 cc. Both cohorts of patients continue to demonstrate greater differences in mean PVR urine volumes from baseline over time in patients with baseline urine volumes greater than 40 cc. The mean differences from baseline are greater in the less than or equal to 40 cc group in both treatment cohorts and the converse is found in the greater than 40 cc group.

## Discussion

In an era which is rapidly becoming defined by increasingly sophisticated treatment planning and radiation delivery techniques, the basic tenant of irradiating what is intended to be treated while respecting normal tissue tolerance has never been more important. To achieve these goals it is necessary to treat a dynamic and moving target, which is exemplified in prostate radiotherapy [14,25]. With dose escalation, strategies must be refined to decrease prostate treatment margins to minimize toxicity to normal structures. Therefore, an investigation of all factors with the potential to influence prostate motion is critical. The bladder and rectum are regarded as the two most important structures in terms of daily prostate motion. This study details the post void residual urine volume prior to daily radiation treatments and the influence of external beam radiation therapy on PVR urine volumes throughout treatment.

If a patient is asked to empty his bladder prior to simulation and then prior to radiation treatment, bladder filling should influence the prostate's position to a lesser degree as previously reported by Zelefsky et al [9]. Although PVR urine volumes were recently explored in cervical cancer treatments, little data is available concerning PVR urine volumes as patients progress through external beam radiation therapy for prostate cancer treated with an empty bladder and in a prone position [22]. Posterior and inferior movement of the prostate gland due to bladder filling was first described by Ten Haken and colleagues, and reproduced by several investigators in subsequent studies [6-8,10]. Melian et al. have reported that bladder filling influenced the position of the prostate in patients treated in the prone position[8]. Zelefsky et al. also demonstrated that bladder volumes greater than 40 cm^3 ^could predict for greater than 3 mm deviations of the prostate and seminal vesicles while in the prone treatment position when the rectal volume is greater than 60 cc [9]. Zellars et al. reported that patients who were treated in the supine position and instructed to have a full bladder prior to treatment demonstrated an associated posterior displacement of the prostate when evaluated 4 to 5 weeks after initiation of therapy [7]. Conversely, other researchers have not seen a relationship between bladder filling and prostate position, although these patients were treated in the supine treatment position [5,15,17].

Bladder filling is more easily controlled on a daily basis than rectal filling, assuming that the patient voids immediately prior to treatment. This strategy is simple and should help to remove the potential influence of the bladder on prostate motion. This paper specifically reports the influence of external beam radiation therapy on serial PVR urine volumes as patients proceed through treatment. Several strategies currently exist for daily image guidance for prostate treatment, therefore the purpose of this paper is not to correlate specific PVR urine volumes with prostate motion, but rather determine the influence of external beam radiation therapy on PVR urine volumes as patients proceed through treatment [25]. If PVR urine volumes remain relatively stable throughout external beam radiation treatment than there would be little correlation to prostate motion from the original planning CT simulation.

Our study population consisted of patients treated with external beam radiation for prostate cancer. Two common types of radiation treatments were studied, pelvic radiation followed by a cone down to the prostate and prostate only radiation. As such the effects of PVR urine volumes could be compared in patients receiving whole pelvic radiation therapy for a portion of their treatment compared to prostate only radiation therapy. These two cohorts provide insight in the potential for PVR urine volume changes in the most common clinical scenarios for definitive external radiation therapy for prostate. Patients in the whole pelvic cohort had larger portions of their bladder irradiated and presumably had the potential for a greater degree of radiation induced bladder irritation.

There were significant differences in the clinical presentation between the two cohorts of patients within the two radiation groups. These differences are attributable to our selection criteria. Importantly, these two groups of patients allowed us to study different treatment strategies, depending on risk of lymph node involvement, on PVR urine volumes as patients progressed through external beam radiation treatment for prostate cancer. Patients treated with prostate only radiation were determined to have lower pre-treatment PSA, lower percent positive biopsies, lower Gleason Scores and clinical stage than patients treated with pelvic radiation. This finding is expected as higher PSA, Gleason Score and clinical stage predicts for a greater probability of lymph node involvement [20]. Our policy was to treat lymph nodes if the risk of involvement was greater than 10%.

The mean individual PVR urine volume over all weeks of treatment in the pelvic and prostate radiation groups was not statistically different with values of 46.3 cc and 50.2 cc respectively. However, mean PVR urine volumes stratified by week in both groups demonstrated the patients treated with whole pelvic radiation had larger baseline PVR urine volumes at the beginning of treatment. Larger baseline PVR theoretically would allows for greater absolute volume changes as radiation induced bladder irritability increased. Although higher baseline mean PVR urine volumes predicted for greater mean PVR urine volumes during treatment, PVR decreased from baseline in both cohorts with the absolute difference within the limits of accuracy of the bladder scanner. Such small differences are unlikely to result in any clinical significance in prostate motion. Also of interest is that the difference from baseline PVR urine volumes in both cohorts appeared to have the greatest change during the first three weeks of treatment and then became well within the limits of accuracy of the bladder scanner. It is likely that patient attention to detail (i.e. bladder emptying) accounted for the changes during the first three weeks of treatment. As such, it is probable that PVR volume determinations early in the course of treatment may be sufficient with subsequent weekly determinations omitted.

Previous research by Zelefsky's group has demonstrated that bladder volumes greater than 40 cc had the potential to influence daily prostate position while treated in the prone position when rectal filling was greater than 60 cc [9]. As such patients were stratified by radiation treatment group and a baseline PVR cutoff of 40 cc. Patients with a baseline PVR = 40 cc did not experience any appreciable change in PVR during treatment while patients with a baseline PVR > 40 cc were most likely to experience changes (i.e. decrease) from the baseline PVR. This marked decline could result in a smaller degree of prostate motion but also in the setup being different from what was initially simulated. Although, patients who are identified with a higher PVR urine volume at the time of simulation may require attention to bladder filling depending on the technologies of daily prostate localization employed. A shortcoming of our study is that patients with substantial decreases in serial PVR's were not re-planned via CT simulation (all patients however were treated with daily cone beam CT guidance).

On average, alpha-blockers were prescribed 4.7 weeks into treatment. Alpha-blockers were not demonstrated to influence PVR in either treatment cohort. This, however, is not surprising since the vast majority of changes in PVR occurred in the first three weeks or therapy. Alpha-blockers were initiated primarily for irritative symptoms.

## Conclusion

External beam radiation therapy results in a clinically insignificant change in weekly post-void residual urine volumes (especially when PVR urine volumes are less than 40 cc), suggesting that radiation induced bladder irritability does not substantially influence bladder residual urine volumes.

## Competing interests

The authors declare that they have no competing interests.

## Authors' contributions

PFO has done statistical analysis as well as drafted the manuscript. GSM has made the selection of patients, involved with the study design, has been involved with writing and revising the manuscript, statistical analysis and final approval of the version to be published. ZAA has been involved with the statistical analysis and design of the tables/figures. WMB has been involved with the statistical analysis. KEW has been involved in manuscript revision and review of the intellectual content. BSK has been involved with statistical analysis. RWG has been involved with statistical analysis and design of the tables/figures. All authors read and approved the final manuscript.
